# Experimental evaluation of the relationship between lethal or non-lethal virulence and transmission success in malaria parasite infections

**DOI:** 10.1186/1471-2148-4-30

**Published:** 2004-09-08

**Authors:** REL Paul, T Lafond, CDM Müller-Graf, S Nithiuthai, PT Brey, JC Koella

**Affiliations:** 1 Unité de Biochimie et Biologie Moléculaire des Insectes, Institut Pasteur, 25 rue du Dr. Roux, 75724 Paris Cedex 15, France; 2 Laboratoire d'Entomologie Médicale, Institut Pasteur de Dakar, 36, Avenue Pasteur BP 220, Dakar, Sénégal; 3 Laboratoire de Parasitologie Evolutive, CC237, CNRS UMR 7103, Université P. & M. Curie, 7 quai Saint Bernard, 75252 Paris Cedex 05, France; 4 Veterinary Parasitology, Faculty of Veterinary Science, Chulalongkorn University, Bangkok 10330, Thailand

## Abstract

**Background:**

Evolutionary theory suggests that the selection pressure on parasites to maximize their transmission determines their optimal host exploitation strategies and thus their virulence. Establishing the adaptive basis to parasite life history traits has important consequences for predicting parasite responses to public health interventions. In this study we examine the extent to which malaria parasites conform to the predicted adaptive trade-off between transmission and virulence, as defined by mortality. The majority of natural infections, however, result in sub-lethal virulent effects (e.g. anaemia) and are often composed of many strains. Both sub-lethal effects and pathogen population structure have been theoretically shown to have important consequences for virulence evolution. Thus, we additionally examine the relationship between anaemia and transmission in single and mixed clone infections.

**Results:**

Whereas there was a trade-off between transmission success and virulence as defined by host mortality, contradictory clone-specific patterns occurred when defining virulence by anaemia. A negative relationship between anaemia and transmission success was found for one of the parasite clones, whereas there was no relationship for the other. Notably the two parasite clones also differed in a transmission phenotype (gametocyte sex ratio) that has previously been shown to respond adaptively to a changing blood environment. In addition, as predicted by evolutionary theory, mixed infections resulted in increased anaemia. The increased anaemia was, however, not correlated with any discernable parasite trait (e.g. parasite density) or with increased transmission.

**Conclusions:**

We found some evidence supporting the hypothesis that there is an adaptive basis correlating virulence (as defined by host mortality) and transmission success in malaria parasites. This confirms the validity of applying evolutionary virulence theory to biomedical research and adds support to the prediction that partially effective vaccines may select for increasingly virulent malaria parasite strains. By contrast, there was no consistent correlation between transmission and sub-lethal anaemia, a more common outcome of malaria infection. However, overall, the data are not inconsistent with the recent proposal that sub-lethal effects may impose an upper limit on virulence. Moreover, clone specific differences in transmission phenotypes linked to anaemia do suggest that there is considerable adaptive potential relating anaemia and transmission that may lead to uncertain consequences following intervention strategies.

## Background

Over the last few decades there has been considerable effort to introduce an evolutionary perspective to biomedical research and examine the extent to which adaptionist argumentation can be used to address questions pertinent to disease management, particularly that of pathogen virulence [1,2]. Contrary to the conventional wisdom that pathogens should evolve to become benign to their hosts (i.e. prudent host exploitation), considerable theoretical work has shown that the extent of pathogen virulence depends on the trade-off between the exploitation of the host (virulence) and the benefits accrued by increasing R_0_, the reproductive rate, either via increasing transmission directly or by reducing host recovery rate [3,4]. Thus, pathogens are expected to evolve a schedule of host exploitation that maximises their transmission and there are an increasing number of experimental studies demonstrating a relationship between transmission and virulence [5-8]. Incorporation of such virulence theory into an epidemiological framework has led to the suggestion that partially effective vaccines targeting pathogen virulence traits (e.g. malaria parasite growth rate) may actually lead to the selection of increased pathogen virulence [9]. Empirical confirmation of such important theoretical predictions requires the examination of how selection may be acting on the virulence factors underlying the pathogenicity through variability in transmission success [10]. Identifying the biological determinants governing the constraint between the transmission/virulence trade-off in turn requires an appreciation of the host-pathogen system in question. This has been recently exemplified by the case of the bacteria *Neisseria meningitides*, where virulence arises through colonisation of atypical tissues with no apparent direct benefit for transmission [11]. However, such apparently short-sighted evolution has been suggested to be an inadvertent consequence of within-host evolution that enables pathogen survival and transmission in diverse host spp. [12]. In these situations, there is an indirect trade-off between virulence and transmission that is strongly influenced by the time lag occurring between the beneficial and deleterious outcomes of within-host evolution [13].

Theoretical advances in virulence theory have revealed the importance of additional properties of host-pathogen systems, such as pathogen population structure and sub-lethal (significantly deleterious for host fitness but not lethal) effects, which can significantly alter the conclusions arising from the simple virulence/transmission trade-off hypothesis [14,15]. Infections are often composed of many simultaneously co-infecting strains that may interact directly or indirectly through their shared host. When parasites share a host, competition for the limited resources is generally expected to favour the most aggressive parasite – the tragedy of the commons [16]. Many theoretical models incorporating selection into an epidemiological framework show that selection favours increasingly virulent genotypes as the number of co-infecting parasite genotypes increases [14,17-20]. The expected levels of virulence, however, depend critically on how parasites interact with their hosts and how co-infecting genotypes affect each other; specifically, optimal levels of virulence depend on the cooperative or competitive nature of host exploitation by co-infecting parasites [15,21-23]. Hence, in what way such co-infection and sub-lethal effects potentially influence virulence evolution needs to be addressed explicitly for each pathogen system. In this paper we address the virulence/transmission relationship for the malaria parasite system and thereby examine the premises underlying the theoretical predictions on vaccine efficacy [9].

Malaria parasites replicate asexually within their vertebrate hosts, but must produce sexual stages (male and female gametocytes) to be transmitted to mosquitoes. Asexual parasites provide the source for gametocyte production and so increasing asexual parasite density is expected to correlate with transmission success. Therefore, the adaptive trade-off hypothesis would predict that faster asexual replication leads both to higher virulence and to higher transmission success [24]. Although transmission success does broadly increase with gametocyte density [25,26], which largely reflects asexual parasite density, many factors can affect transmission success [27]. I.e. high density does not always guarantee infection success [25,28]. Malaria parasites demonstrate considerable phenotypic plasticity in the production of sexual transmission stages. This plasticity appears to be linked to the quality of the blood environment: as conditions for asexual replication worsen, the parasite alters its sex allocation strategy by changing the sex ratio of gametocytes [29] or the proportion of asexual parasites producing gametocytes [30-32]. Thus transmission traits (e.g. gametocyte density and sex ratio) are highly susceptible to environmental changes and may therefore not conform to the simple trade-off between transmission and mortality. Although infection with malaria parasites can lead to host death, non-lethal anaemia is more commonly the result, where the parasitic infection provokes a reduction in haematocrit (red blood cell density) [33] and thus a reduced quality resource for parasite replication. Thus, anaemia is both a physical manifestation of virulence and a trigger of sex allocation.

In this study we examine the trade-offs between transmission and virulence defined by mortality and anaemia in single and mixed clone infections, using the avian malaria system *P. gallinaceum *in the chicken host. In this specific system, anaemia and parasite transmission are linked physiologically, where the host's response to anaemia (reticulocyte production) triggers an increase in the proportion of gametocytes that are male rather than female [29]. This has been proposed to be an adaptive parasite response to assure mating success in the face of an immune response (transmission-blocking immunity) that develops simultaneously to the anaemia [34-37]. Experimental abnegation of this immune response can be achieved by injection of a high density parasite inoculum, which results in an uncontrolled infection, host death, and importantly, an absence of both the host reticulocyte response to anaemia and the linked parasite sex allocation response [37]. Thus, we are able to generate infections known *a priori *to result in host death or control by the host, enabling independent study of parasite transmission to mosquitoes with respect to either mortality or anaemia. Under both infection conditions, we examine the impact of mixed clone infections on overall virulence and transmission. Thus we assess to what extent there is an adaptive basis relating virulence and transmission under infection conditions of theoretical significance.

## Results

### Course of infection – competition between clones

**Figure 1:**
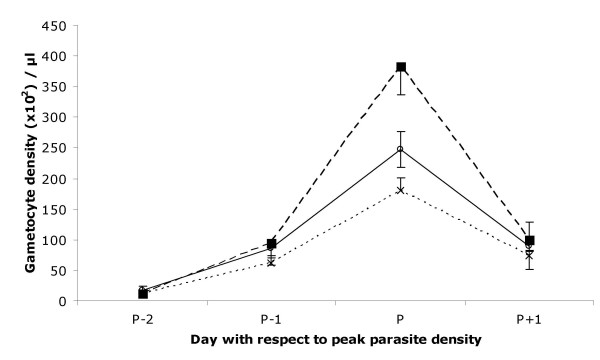
*P. gallinaceum *gametocyte (sexual) parasite density (mean ± S.E.) following intra-muscular injection of 10^6 ^parasites single clones or 5 × 10^5 ^of each clone in mixed infections. *Thai *clone (square, long dotted line); *SL *clone (open circles, solid line); mixed clones (cross, dotted line).

**Figure 2:**
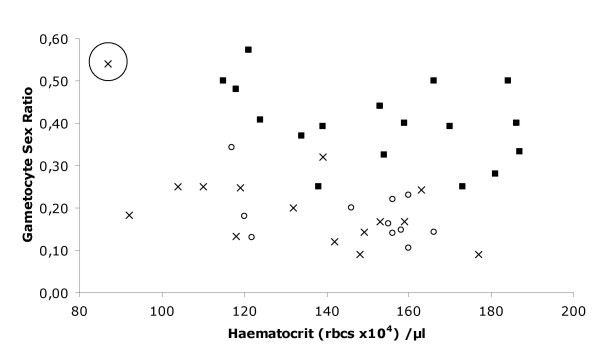
Gametocyte sex ratios (proportion male) of mixed *vs*. single clone infections compared with the corresponding haematocrit (**r**ed **b**lood **c**ell density) measured on the same day of infection. Each individual infection was measured daily for both these parameters until either the host died or the infection was cleared by the host (thus until the peak of infection). Infections were initiated by injection of 10^6 ^parasites for single clone treatments and 5 × 10^5 ^of each clone in mixed infections. *Thai *clone (squares); *SL *clone (open circles); mixed clones (crosses). Circled cross represents one individual that had a high sex ratio associated with a high density of reticulocytes on one particular day (see text).

We first considered infection by injection of 10^6 ^infected red blood cells (iRBC) (an inoculum that almost always leads to a lethal infection outcome) of either of two clones of *P. gallinaceum *(the *Thai *or *SL *clone) or an equal mix of the two. The peak densities of asexual parasites did not differ between *Thai *and *SL *clone infections (χ^2^_1 _= 0.3 P = 0.629), but were both significantly higher than those in mixed clone infections (combined single clones vs. mixed clone χ^2^_1 _= 6.3 P = 0.012). The peak gametocyte density was, however, significantly higher in *Thai *than in *SL *clone infections (χ^2^_1 _= 4.3 P = 0.038), and higher in both the single clone groups than in the mixed infections (χ^2^_1 _= 4.05 P = 0.044) (Fig. 1). The extent of anaemia reflected parasite density, but was greater in the mixed clone group than in either of the two single clones, despite its lower parasite density (parasite density χ^2^_1 _= 14.1, P < 0.001; Group χ^2^_2 _= 6.1, P = 0.048). This effect of co-infection was confirmed with a comparison of the mixed clone with the 2 single clone type infections grouped together (parasite density χ^2^_1 _= 14.2, P < 0.001; combined single clones vs. mixed clone: χ^2^_1 _= 5.8, P = 0.016). The gametocyte sex ratios (proportion of gametocytes that were male) were found to be different among groups (χ^2^_2 _= 33.5, P < 0.001), where the *Thai *clone had a higher sex ratio than the *SL *clone infections. Closer inspection and analysis of the data revealed similar sex ratios in the *SL *clone and mixed clone infections (Fig. 2) (Haematocrit χ^2^_1 _= 16.8, P < 0.001; Group χ^2^_1 _= 25.1, P < 0.001). As previously observed after infections by injection with high inocula [37], the production of reticulocytes is almost always absent during the growth phase of the infection that is followed either immediately by death or "crisis" when the host destroys all red blood cells (and parasites) and floods its system with reticulocytes. Notably, here one individual displayed a strong reticulocyte response to anaemia during the growth phase of infection, as occurs in natural, low density infections (See Methods); this was predictably associated with a strong increase in sex ratio (see Fig. 2 circled cross) [29].

These data strongly suggest that the clones had a negative impact on each other, thus resulting in lower densities of asexual parasites and hence gametocytes. The anaemia (and hence sub-lethal virulence) was greater in the mixed clone infections, as previously observed in mouse models of malaria [38] and natural infections in humans [39], but was not accompanied by an increase in parasite densities. Thus, such a virulence outcome likely reflects a more intense host response to infection rather than an adaptive response relating pathogenicity and transmission [10].

**Figure 3:**
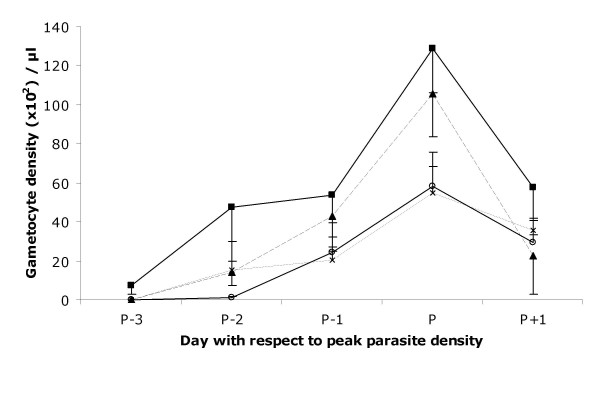
*P. gallinaceum *gametocyte (sexual) parasite density (mean ± S.E.) following infection by either or both parasite clones using a fixed number of mosquitoes. *Thai *or *SL *clone alone, 8 infectious mosquitoes for either clone per bird; mixed infections were initiated using 8 infectious mosquitoes of each clone per bird (8+8) or 4 infectious mosquitoes of each clone per bird (4+4). 3 birds were thus infected for each treatment type and the experiment was performed twice using different batches of mosquitoes infectious for each clone. *Thai *clone (squares, thin line); *SL *clone (open circles, thick line); 8+8 mixed clones (triangles, long dotted line); 4+4 mixed clones (crosses, short dotted line).

**Figure 4:**
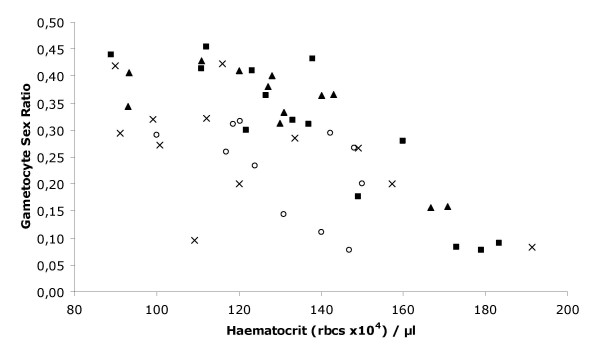
Gametocyte sex ratios (proportion male) of mixed *vs*. single clone infections compared with their corresponding haematocrits (**r**ed **b**lood **c**ell density) measured on the same day of infection. Each individual infection was measured daily for both these parameters until either the host died or the infection was cleared by the host (thus until the peak of infection). *Thai *or *SL *clone alone, 8 infective mosquitoes per bird; Mixed infections were initiated using 8 or 4 infective mosquitoes from batches of mosquitoes infected with either of the clones. *Thai *clone (squares); *SL *clone (open circles); 8+8 mixed clones (triangles); 4+4 mixed clones (crosses).

Secondly we considered the outcome of infections initiated by infected mosquito bites (i.e. as with natural infections). These sporozoite-induced infections were initiated by gorging a fixed number of infected mosquitoes on the individual birds: 8 mosquitoes per bird for the single clone infections and either 8+8 or 4+4 for the mixed clone infections. Thus we controlled for total or clone-specific dose in the mixed infections. The experiment was performed twice, with a different batch of infected mosquitoes used for each experiment. The resulting infections were very consistent and no differences were found between the two experimental replicates for any of the analyses. Parasite densities of the *Thai *clone were significantly higher (χ^2^_1 _= 4.73, P = 0.029) than the *SL *and mixed clone infections (4+4 or 8+8), which did not differ from one another. Gametocyte densities followed the patterns of asexual parasite densities, except for the high mixed clone infection (8+8), whose peak gametocyte production paralleled that of the *Thai *clone (Fig. 3). Again, the extent of anaemia reflected parasite densities and treatment group (Interaction between parasite density and group χ^2^_3 _= 7.9, P = 0.048). Data inspection and model simplification regrouping the treatments into single *vs*. mixed clone infections revealed that mixed clone infections resulted in greater anaemia for a given parasite density (Parasite density χ^2^_1 _= 52.8, P < 0.001; mixed *vs*. single clone infections χ^2^_1 _= 6.8, P = 0.009). This increased anaemia was particularly evident for the 4+4 mixed infection group and was reflected in the significantly stronger reticulocyte response in this group (mixed *vs*. single clone infections χ^2^_1 _= 6.3, P = 0.012). Once again, the gametocyte sex ratio varied with group and anaemia independently (group: χ^2^_3 _= 11.7, P = 0.008; haematocrit: χ^2^_1 _= 16.2, P < 0.001) (Fig. 4). Data inspection and model simplification showed that *Thai*/8+8 type infections had a significantly higher sex ratio (χ^2^_1 _= 10.7, P = 0.001) than *SL*/4+4 types and both types responded similarly to haematocrit (χ^2^_1 _= 17.7, P < 0.001) (Fig. 4).

Thus, again the data support the notion that there is competition between the clones. The greater anaemia in mixed infections was again observed. In addition, the level of anaemia and the reticulocyte response to infection were greatest for the lowest inoculation dose. The extent of anaemia did not, however, seem to affect parasite densities in the mixed infections, where the parasitological parameters measured (e.g. asexual parasite / gametocyte densities and the sex ratio) aligned with those of either one of the 2 clones. Although the clones do appear to compete aggressively, sub-lethal virulence was more strongly influenced by infecting dose. Thus the sub-lethal measure of virulence (i.e. anaemia) appears to be more strongly dependent on the host's response to infection than on parasite density *per se*.

### Virulence and transmission success to mosquitoes

**Figure 5:**
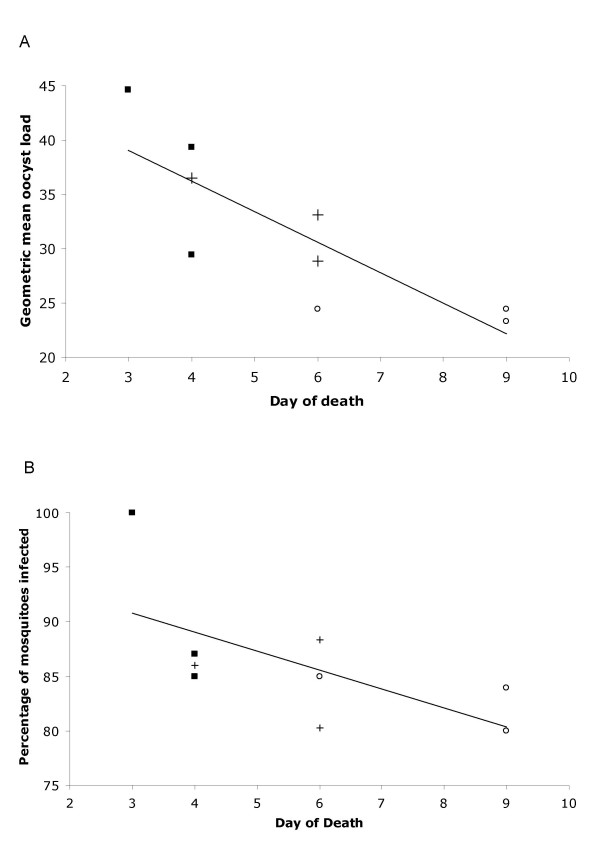
Transmission success as measured by (**A**) the geometric mean oocyst load per mosquito and (**B**) the percentage of mosquitoes that were positive for at least one oocyst, related to day of death for the 2 single clone and the mixed clone type infections. Three birds in each group (injected with single or mixed parasite clones as in Figs. 1 & 2) were gorged on mosquitoes on the first day of patent infection. *Thai *clone (squares); *SL *clone (open circles); mixed clones (crosses). Line represents least squared residual best fit for the relationship between oocyst load and day of death. (**A**) R^2 ^= 0.68, P = 0.017; (**B**) R^2 ^= 0.41, P = 0.032.

For the iRBC-induced infections, one set of mosquito transmission studies was carried out on individuals with comparable parasite and gametocyte densities 3 days after inoculation, when parasite densities were low. Transmission success to mosquitoes at this early infection time point, as measured by both geometric mean oocyst load and the percentage of mosquitoes that were oocyst positive, was proportional to the subsequent rate of death (oocyst load: χ^2^_1 _= 5.6, P = 0.017; percentage infected: χ^2^_1 _= 4.6, P = 0.032) (Figs. 5a &5b). Infection type (*Thai *vs. *SL *vs. mixed clone) was not found to correlate with transmission success (oocyst load: χ^2^_2 _= 2.3, P = 0.339; percentage infected: χ^2^_2 _= 3.34, P = 0.17). In addition, neither sex ratio (oocyst load: χ^2^_1 _= 2.5, P = 0.114; percentage infected: χ^2^_1 _= 2.1, P = 0.15) nor gametocyte density (oocyst load: χ^2^_1 _= 0.1, P = 0.75; percentage infected: χ^2^_1 _= 0.3, P = 0.62) was correlated with transmission success. This is evidence that virulence, as defined by mortality, is positively correlated with transmission success.

**Figure 6:**
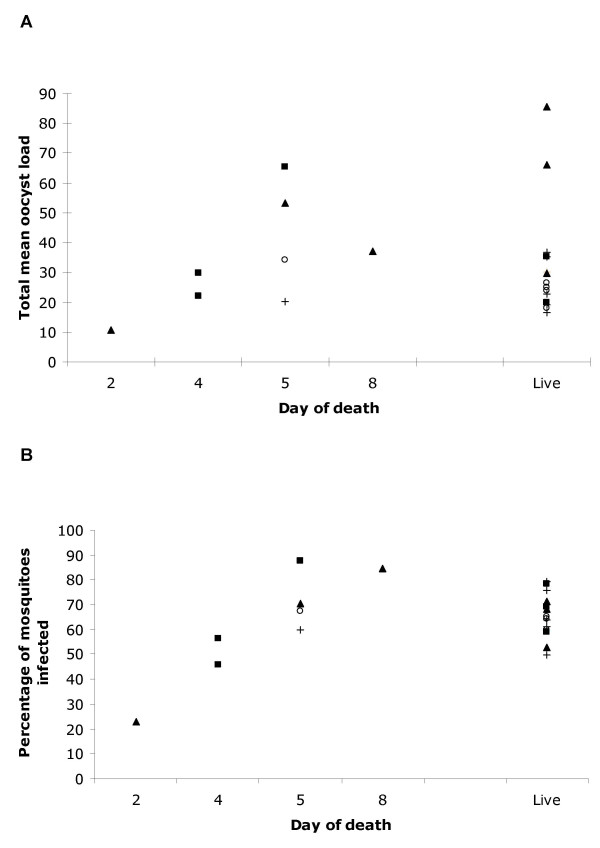
Comparison of lifetime transmission success with day of host death. Lifetime transmission success is defined as (**A**) the mean number of oocysts per mosquito for a given day totalled over the 4 days of acute phase transmission; (**B**) the percentage of mosquitoes with at least one oocyst. Infections were initiated using infectious mosquitoes as in Figs. 3 & 4. *Thai *or *SL *clone alone, 8 infective mosquitoes per bird; Mixed infections were initiated using 8 or 4 infective mosquitoes from batches of mosquitoes infected with either of the clones. Mosquitoes were gorged on all birds from the first day parasites were patent in the blood, (a) *Thai *clone (square); *SL *clone (open circles); mixed 8+8 clones (triangle); mixed 4+4 clones (crosses).

For the sporozoite-induced infections (Figs. 6a &6b), we first carried out the same analysis as above, comparing transmission success to mosquitoes at the earliest infection time point with the rate of death, using only those 8 individuals that died. The percentage of mosquitoes infected was proportional to the rate of death (χ^2^_1 _= 4.54, P = 0.032), however the oocyst load was not (χ^2^_1 _= 3.36, P = 0.067). By contrast, infection type was not found to correlate with the percentage infected (χ^2^_3 _= 5.56, P = 0.135), but did with the oocyst load (χ^2^_3 _= 9.71, P = 0.021); this latter result is most likely the consequence of the small sample size (n = 8), three individuals of which were infected with the *Thai *clone, died on days 4 and 5 and produced very similar oocyst loads in the mosquitoes. The significant correlation between rate of death and the percentage of mosquitoes infected in both iRBC- and sporozoite-induced infections, despite their small sample sizes, provides consistent evidence that virulence, as defined by mortality, is positively correlated with transmission success.

For the sporozoite-induced infections, we then examined the relationship between mortality and parasite lifetime transmission success, as defined by total mean geometric oocyst load and total percentage of mosquitoes infected. For these analyses, individuals were first grouped according to whether they lived or died, and secondly, if they died, whether it was before or after the 4-day acute transmission period. There was no significant difference in either the percentage of mosquitoes infected (χ^2^_1 _= 1.91, P = 0.16) or the total oocyst load (χ^2^_1 _= 0.2, P = 0.63) between those individuals that died at any time and those that lived. However, those individuals that survived for the 4-day acute transmission period, irrespective of whether they subsequently died, infected a higher percentage of mosquitoes (percentage infected: χ^2^_1 _= 12.9, P < 0.001) and with a higher mean oocyst density mosquitoes (χ^2^_1 _= 18.2, P < 0.001) than those 3 individuals that died rapidly. Excluding these 3 early deaths, we compared the transmission success of those that lived and died following the acute transmission period. There was no significant effect of host mortality on either the percentage of mosquitoes infected (χ^2^_1 _= 2.37, P = 0.12) or on mean oocyst loads (χ^2^_1 _= 1.07, P = 0.33). These results suggest that although parasite-induced mortality may incur a transmission cost by reducing the duration of transmission, there is no additional effect of mortality on transmission success. This absence of a positive relationship between virulence and transmission success contradicts the results in the previous paragraphs where the rate of mortality was correlated with a single day measure of transmission success. This may, however, be the consequence of the small sample size (number of individuals that died after completing the acute transmission period, n = 5) and complexity of infection types.

**Figure 7:**
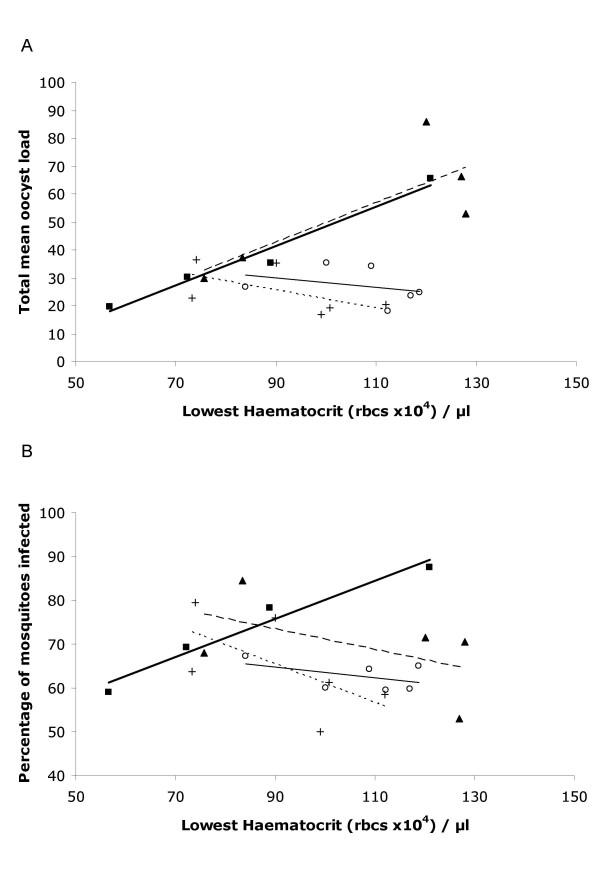
Comparison of lifetime transmission success with peak anaemia. Lifetime transmission success is defined as (**A**) the mean number of oocysts per mosquito for a given day totalled over the 4 days of acute phase transmission; (**B**) the percentage of mosquitoes with at least one oocyst. Infections were initiated using infectious mosquitoes as in Figs. 3, 4 & 6. *Thai *or *SL *clone alone, 8 infective mosquitoes per bird; Mixed infections were initiated using 8 or 4 infective mosquitoes from batches of mosquitoes infected with either of the clones. Mosquitoes were gorged on all birds from the first day parasites were patent in the blood. Individuals that died before completion of the transmission period are excluded (a) *Thai *clone (square – thin solid line); *SL *clone (open circles – thick solid line); mixed 8+8 clones (triangle – long dotted line); mixed 4+4 clones (crosses – short dotted line). Lines are least squared residual best fit.

Lifetime transmission success was then compared against the level of anaemia at infection peak (Figs. 7a &7b). Because mixed clone infections led to increased anaemia independent of parasite density, we first examined the transmission/anaemia relationship in the single clone infections. There was a strong interaction between clone type and haematocrit on lifetime transmission success for both the percentage of mosquitoes infected (χ^2^_1 _= 12.2, P = 0.0005) and the total mean oocyst load (χ^2^_1 _= 10.21, P = 0.0015). In the *Thai *clone infections, anaemia at infection peak was negatively correlated to total mean oocyst load (χ^2^_1 _= 7.28, P = 0.007) and the percentage of mosquitoes that were infected (χ^2^_1 _= 8.4, P = 0.004); in the *SL *clone infections, however, there was no correlation between anaemia and either oocyst load (χ^2^_1 _= 0.85, P = 0.36) or the percentage of infected mosquitoes (χ^2^_1 _= 0.56, P = 0.46). When examining the data for all infection types, although the 8+8 mixed clone infections aligned with the *Thai *clone infections when considering the oocyst load, this was lost when considering the percentage of mosquitoes infected (Figs. 7a &7b). Why there is this disparity in mosquito infection parameters for this mixed infection group is unclear, but highlights the importance of considering mixed infections and of careful choice of the measure of transmission success used. Further work on transmission success from mixed infections is clearly required. In conclusion, the data suggest that there are clone-specific effects relating sub-lethal virulence to transmission and importantly that in the case of the *Thai *clone, that excessive anaemia may be a fitness cost for virulence evolution.

## Discussion

This study examined the validity of some basic assumptions underlying evolutionary models of virulence for the specific case of malaria parasites, and hence the extent to which such models may be relevant to malaria control. One of the fundamental assumptions is that a parasite's virulence is a necessary consequence of the strategy that it uses to exploit its host in its (evolutionary) attempt to achieve maximal transmission. For the case of malaria, the production of transmission stages (gametocytes) depends on the asexual replication of the parasite clone within the host's blood system. Thus, faster asexual replication would be expected to result in more transmission before the host's immune responses control the infection, but also to cause greater host damage and to increase the risk of the host's death. Hitherto, experimental studies have either used gametocyte density as a proxy for transmission success [24], or taken a snap-shot count of successfully transmitted parasite stages at a time of estimated maximal transmission [7,40,41]. Here we measured transmission success directly by gorging mosquitoes on the infected hosts and counted the number of successfully transmitted parasites either at a time independent of the virulence outcome or throughout the period during which the parasite transmits from the vertebrate host to the mosquito vector. We found (i) that in both iRBC and sporozoite-induced infections, transmission success was positively correlated with host mortality rate but (ii) that there was a total transmission cost to host death only if the host died before the completion of the transmission period; if the host died after completing the acute transmission period there was no impact on total transmission. Finally (iii) there was no clear correlation between transmission success and anaemia (sub-lethal virulence): infections with one of the parasite clones resulted in a negative virulence-transmission relationship whereas with the other clone there was no relationship. These observations suggest that although malaria parasites may conform to the predictions of virulence theory based on parasite-induced host mortality, there appears to be no general trade-off between transmission and sub-lethal virulence. Notably, measurable parasitological parameters such as asexual and sexual parasite densities were predictive of neither mortality rate nor transmission success. This is because the relationships between asexual density and host mortality and between transmission success and the densities of asexual stages and gametocytes are not straightforward and are influenced considerably by developing immune responses. Indeed, despite the appealing simplicity of the idea, it is not obvious that there should be simple measurable parameters, such as asexual parasite density, defining parasite virulence or that these should correlate in a straightforward manner with transmission; this has been discussed in depth for the case *Neisseria*, as noted in the Background section [11,12]. Irrespective, however, of the precise biological details underlying parasite-induced mortality, malaria parasites do generally confirm one of the most robust predictions of virulence theory. Therefore, natural parasite populations might confirm the prediction that, in the simplest case of single clone infections in a homogeneous host population, intervention strategies targeting asexual replication rate could select for increased virulence [9].

Previous work in experimental mouse models found there to be positive genetic correlations between virulence, asexual density and transmission [24]. However, sub-lethal effects, rather than lethal infection outcome, were found to impose an upper threshold to virulence [7] and transmission was maximised at intermediate levels of host morbidity [42]. In our system, host death did reduce total transmission success, but only when the mortality rate was very high; mortality *per se *incurred no cost to transmission. In addition, more virulent infections were generally more infectious prior to host death. These results conform to standard virulence theory where there is a relationship between transmission and virulence, such that intermediate levels of virulence evolve. By contrast, the relationship between transmission success and sub-lethal virulence (anaemia) was less clear. On the one hand, infections with one of the parasite clones (*Thai*) confirm the suggestion from the mouse models [42] that sub-lethal effects impose selection against virulence. On the other hand, there was no transmission/virulence relationship for the other clone. How to interpret such results? In the mouse models [42], sub-lethal virulence was measured as a composite parameter including weight loss and anaemia; here we only used anaemia and therefore may have missed additional negative effects of the parasite on the host that might alter our conclusions. Indeed, for a given parasite dose, the level of anaemia induced was the same for the two clones. Thus, there was apparently no between-clone variance in putative parasite traits provoking anaemia, but there was clone-specific variation relating transmission to sub-lethal virulence. Additional clone-specific virulent effects on the host (e.g. weight loss) may therefore be as important as anaemia. An additional consideration is the proximate effect of anaemia on transmission. Previously, clone *SL *has been shown to adaptively alter its gametocyte sex ratio according to the host response to anaemia [29]. Notably the gametocyte sex ratio of the *Thai *clone was different from that of the *SL *clone, though its response to anaemia appeared similar (sex ratio became less female-biased). If the two clones differ in a transmission trait (sex ratio) that is apparently sensitive to host anaemia, they may be expected to vary in other, less evident traits implicated in transmission. Such variation in transmission traits would result in between-clone variability in the anaemia/transmission relationship. Further exploration of the covariance in sex ratio and the anaemia/transmission relationship is clearly required to ascertain whether sex ratio can be used as a marker phenotype for the complex interactions between the parasite and the host that determine parasite transmission success. In conclusion, the data are not inconsistent with the proposal that sub-lethal effects may impose an upper limit on virulence [42], but a more detailed understanding of parasite traits implicated in transmission is required.

The final consideration concerns how the complexity of infection may alter the virulence/transmission trade-off. Competition between clones in multiple infections would generally be expected to favour the more virulent clone [20], although sub-lethal effects can considerably reduce the optimum level of virulence [15]. Malaria parasites are confronted with an unpredictable number of co-infecting clones. Under such conditions, parasites may employ facultative strategies of host exploitation according to co-infecting clone number and virulence would be expected to increase in mixed *vs*. single clone infections [38]. Anaemia was increased in all mixed infections, as previously observed in mouse models [38]. If parasites have evolved transmission strategies correlating with anaemia, such increased anaemia would be expected to result in altered transmission success. Overall transmission success was, however, unaltered and consistent with that observed for the single clone infections. However, the degree of increased anaemia in the mixed clone infections did not seem sufficient to alter the gametocyte sex ratios, previously shown to respond adaptively to changes in anaemia [29]. Thus, the sub-lethal virulence effect of the mixed infection does not seem to be sufficient to reduce the efficacy of the parasite's transmission strategy, of which facultative shifts in sex ratio is but one manifestation. In addition, although molecular data were not available, transmission phenotypes (e.g. gametocyte density and sex ratio) of mixed clone infections seemed to align themselves with either of the two clones: i.e. there seemed to be competitive dominance by one clone of the other, although the genetic identity of the clones abnegated any objective measurement. Interestingly, the competitive outcome was seemingly resolved early on in the infection. This suggests that there are clone-specific biological features determining the outcome at the very early stages of infection. The importance of the early stages of infection in the host-parasite interaction was further highlighted by the positive correlation between transmission success at the start of infection and host mortality rate, which occurred many days later.

An additional surprising outcome of the mixed infections was that the identity of the dominant clone depended upon infecting dose. Infecting dose has previously been shown to influence disease severity in mouse models, but importantly there were consistent clone-specific differences in disease severity across a wide-range of doses [43]. Here, it is notable that at high infection doses (iRBC-induced infections) there were no differences between peak parasite densities of the two clones whereas at low infection doses (sporozoite-induced), the *Thai *clone reached a higher peak density. Thus we found dose-dependent differences that may not only alter the potential competitive outcome of a mixed infection, but may also alter the relative virulent nature of the 2 clones: i.e. relative differences in maximum parasite densities of clones may be more influenced by infecting dose than by clone identity. Our data from two clones are clearly limited, but if infecting dose is important, there may be additional subtle consequences of local heterogeneity in transmission intensity (number of infectious mosquito bites per host per unit time; i.e. dose) in addition to increasing R_0 _[44,45] and genetic complexity [46].

## Conclusions

Research using animal models is generating increasing evidence supporting an adaptive basis to life history traits and virulence of malaria parasites [7,24,42]. The studies presented here generally confirm these previous findings from a mammalian model system. Where they are different, probably reflects fundamental life history strategies pertaining to blood use (e.g. type of red blood cell invaded) [47], which include strategies for transmission stage production [30,34,35]. Such life history traits are well-documented (reviewed in [47]) and may be simple phenotypic markers reflecting the suite of alternative strategies available to the parasite. While the relevance of laboratory models to natural systems is easily criticised, the consistency of infection patterns across both avian and mammalian models suggests that there are general features of *Plasmodium-*host interactions. Therefore, with careful consideration of the biological details of each system, extrapolation from laboratory model to field data may be justified. Where field data fail to fit predictions, it is likely that we are missing important biological processes underlying the system in question [48]. One major deficiency of models is the inability to simulate repeated infections. With rare exceptions [49,50], the emphasis is placed on primary infections. The majority of infected humans, however, are not presenting with their first infections and will have confronted parasites for a considerable length of time. The host immune response to primary *vs*. re-infections is markedly different and it is likely that the host haematological response alters as well. The proportion of individuals confronting parasites for the first time (i.e. primary infections) depends on the transmission intensity, which also determines the parasite population structure and the parasite dose. Thus, interpreting field patterns with respect to evolutionary predictions necessitates stratification according to the epidemiology of the populations in question. However, despite such complexity, that both laboratory and field populations of malaria parasites generally conform to evolutionary theories concerning life history traits (e.g. sex allocation) and virulence is encouraging, and suggests that evolutionary theory can play an important role in predicting consequences of public health intervention strategies [9]. It remains, however, to be seen whether less conventional, but probably more important, measures of parasite virulence such as anaemia can be considered within an evolutionary framework.

## Methods

### Parasite, host and mosquito species

Two strains of the chicken malaria parasite *P. gallinaceum *were used: Strain 8A (originally from Sri Lanka and obtained from D. Kaslow, NIH, Bethesda, USA) and a new Thai strain (obtained from S. Nithiuthai, Chulalongkorn University, Bangkok, Thailand). In this paper, 8A strain is designated as *SL *and the Thai strain as *Thai*. Both strains were cloned with limiting dilution in 5-day old chick hosts (*Gallus gallus domesticus*) (INRA, France). The clones were maintained *in vivo *by inoculation of 1 ml infected blood (20–45% parasitaemia, percent red blood cells (rbcs) infected) into naïve chicken hosts, with frequent passage through the mosquito vector, *Aedes aegypti *(Liverpool Blackeye strain). For experimentation, different, healthy 3-week old White Leghorn chickens were used for each experiment. All experimental animals were maintained according to European Union guidelines. Parasitaemias were obtained with Giemsa staining of daily blood smears. Parasitaemias and reticulocytes were calculated as percentages observed in a minimum of 100 rbcs; gametocytaemias observed in 10,000 rbcs. Haematocrit (number of rbcs per unit volume) was measured daily for each host using a haemocytometer. Thus, parasite and gametocyte densities are the number of rbcs infected with any parasites or gametocytes per unit volume. Mature male and female gametocytes are distinguishable after Giemsa staining: males stain a pale rose with no distinct nucleus, females stain blue with a distinct red nucleus [51,52]. Sex ratios based on counts of 50–75 gametocytes were found to be representative. We calculated sex ratios from the lesser of 50,000 rbcs or 100 gametocytes. Sex ratios are given as the proportion of males.

*Ae. aegypti *mosquitoes were used in all transmission studies. Mosquitoes were maintained under standard conditions (80% humidity and 26°C). Transmission success was measured by (i) the percentage of mosquitoes positive for oocyst stage parasites and (ii) the mean oocyst density in gorged mosquitoes. Oocysts are the zygote stages of the parasite found on the mosquito stomach wall and which are those parasites that have developed successfully from fertilised female parasite gametes (gametocytes in the vertebrate host give rise to gametes once inside the mosquito bloodmeal which then undergo fertilisation; only fertilised gametes can continue development). Oocyst stage parasite counts were made 7 days post mosquito infection on midguts dissected from 30 gravid females and then stained with 0.5% mercurochrome in 1× Phosphate Buffer solution. Mean oocyst number per mosquito was chosen as an additional measure of transmission success to the number of infected mosquitoes for several reasons. Firstly, oocyst number is a more sensitive measure and is related to the percentage of infected mosquitoes by a simple negative binomial relationship [53]. Secondly, subsequent transmission from an infected mosquito to a new host may be affected by overall oocyst number through altering the number of sporozoites injected during a bloodmeal (See [47] for relevant literature).

### Experimental design

a) Infection was initiated by injection of parasitized blood to generate an uncontrolled infection (in this paper, these infections are denoted iRBC-induced). Chickens were inoculated by intra-muscular injection of 10^6 ^parasites: 6 were inoculated with the *SL *clone, 6 with the *Thai *clone and 6 with 5 × 10^5 ^of each clone. *Ae. aegypti *mosquitoes were gorged upon 3 infected chickens from each group, chosen for their matching parasite densities on the day *P. gallinaceum *was detectable by blood smear (>0.1% parasitaemia). Transmission success (mean number of oocysts per mosquito) on day 3 post inoculation, when parasites were visible in the blood smear (>0.1%), was subsequently related to the day at which that individual died.

b) Infection was performed using infectious mosquitoes to mimic natural low intensity infections (sporozoite-induced infections). Experimental infections were induced by gorging infected mosquitoes on the chicken hosts. 4 experimental groups (3 chickens per group) were considered: (i) 8 infective bites per host using the *SL *clone (ii) 8 infective bites per host using the *Thai *clone (iii) 4 infective bites from the *Thai *clone and 4 infective bites from the *SL *clone per host (iv) 8 infective bites from the *Thai *clone and 8 infective bites from the *SL *clone per host. The presence of sporozoites in each of these mosquitoes was verified subsequently by dissection of the salivary glands. (Note: the clones do not differ in the gene sequences of the molecular markers available for this species in Genbank (NCBI), so that the clones could not be differentiated and identified, unpubl. data). This experiment was repeated using different batches of mosquitoes infected with either parasite clone and healthy, uninfected chickens. Within each experiment for each parasite clone, each individual was infected by mosquitoes coming from the same cage.

*Ae. aegypti *mosquitoes were gorged upon all infected chickens on the 1st day *P. gallinaceum *became patent in the blood (<0.1% parasitaemia), predictably day 7 (following the 7 day pre-erythrocytic developmental period), and for the next 3 days at which point the infection reaches its zenith; this is the acute stage of infection. Post-peak infection the parasite is rapidly cleared by the chicken and resurges only intermittently at very low densities. This chronic stage of infection represents the infectious reservoir yielding irregular low levels of transmission to mosquitoes [54]. The initial acute phase results in very high transmission rates and is here taken to represent the parasite lifetime transmission success (total of the daily mean number of oocysts per mosquito) and is compared against the highest level of anaemia (lowest haematocrit) observed in each individual chicken.

### Statistical Analyses

Statistical analyses were conducted using the statistical package Genstat 6.1. Because each individual chicken was included in the data set many times, we corrected for repeated measures by fitting a generalised linear mixed model (GLMM procedure), nesting day within individual chicken in the random model. Parasite densities and blood cell counts were analysed specifying a Poisson error structure.

Transmission success: the percentage of mosquitoes that were infected was analyzed using logistic regression specifying a binomial error structure with a logit link function and the mean oocyst densities were analyzed with a logistic regression specifying a Poisson error structure with a logarithmic link function (which gives the same fit as a negative binomial, [55]). For the iRBC-induced infections, transmission success was analyzed with respect to day of death, sex ratio and gametocyte density. For sporozoite-induced infections, transmission success on the first day of infection was similarly analyzed with respect to day of death for those individuals that died. In addition, we examined whether host death incurred a transmission cost by comparing transmission success of (i) the bird hosts died or not and (ii) whether birds survived the acute transmission period or not. Between-group comparisons of parasite lifetime transmission success for each individual were also analyzed with respect to the level of anaemia at infection peak.

For the sex ratio and transmission analyses the data were over-dispersed and so were corrected for by estimating a dispersion parameter for each analysis. Statistical significance was presented as Wald statistics, which have a χ^2 ^distribution. When sex ratio was used as an explanatory variable it was arcsine-transformed.

## Author's contributions

RELP carried out the experimental studies, TL assisted in iRBC-induced studies, CMG assisted in experimental design, NS isolated the Thai strain parasite, PTB assisted in experimental design and edited the manuscript and JCK in elaboration of the manuscript.
